# Applying local epidemiological data to national policy – the case study of the epidemiology of wrist and hand injury in Jerusalem

**DOI:** 10.1186/s13584-019-0323-7

**Published:** 2019-06-05

**Authors:** Moran Bodas, Adi Givon, Israeli Trauma Group, N. Abbod, H. Bahouth, M. Bala, A. Becker, M. Ben Eli, A. Braslavsky, I. Grevtsev, I. Jeroukhimov, M. Karawani, B. Kessel, Y. Klein, G. Lin, O. Merin, Y. Mnouskin, A. Rivkind, G. Shaked, D. Soffer, M. Stein, M. Weiss, Kobi Peleg

**Affiliations:** 1 Israel National Center for Trauma & Emergency Medicine Research, The Gertner Institute for Epidemiology and Health Policy Research, Sheba Medical Center, Tel Hashomer, Ramat-Gan, Israel; 2 The Department of Emergency Management & Disaster Medicine, School of Public Health, Sackler Faculty of Medicine, Tel-Aviv University, Tel Aviv-Yafo, Israel

**Keywords:** Wrist and hand injury, Trauma, Epidemiology, Jerusalem, Israel

## Abstract

Recently, Luria et al. (2019) published a paper in *The Israeli Journal for Health Policy Research* describing the epidemiology of wrist and hand injuries in two hospitals in Jerusalem. In this important paper, the authors were able to identify two subpopulations at higher than average risk for such injuries.

It should be noted that local epidemiological findings could differ from findings for regional, national and international settings. Therefore, it is important to explore the extent to which these findings can be further generalized to other contexts, especially when considering health policy changes.

In this commentary, we explore this notion by comparing the results of the Jerusalem Study to those obtained from the Israel National Trauma Registry for the same period. The findings suggest that extrapolating the local findings to the national level should be done cautiously, in light of various differences that were observed.

## Background

In a recent paper published in *The Israeli Journal for Health Policy Research* by Luria et al. [4], hereinafter the “Jerusalem Study”, the authors describe the epidemiology of wrist and hand injuries from two hospitals in Jerusalem. The paper reports on a survey which included a final sample of 799 patients from a total of 1294 who were treated for wrist and hand injuries between April and June 2013. Participants in the study were asked to provide details pertaining to their injury patterns and circumstances. Demographics were collected to assess differences in injury epidemiology across groups. In the conclusion of this important paper, the authors identified two subpopulations at higher than average risk for wrist and hand injury, namely: (a) non-ultra-orthodox Jewish women over the age of 65, which were at higher risk for contusions, and (b) Ultra-orthodox Jews under the age of 10 and Muslim teens, which were at higher risk of crush injuries.

While these findings are clearly important for the planning and management of health care services in Jerusalem, it is important to note that extrapolating from local epidemiological findings to other levels, i.e. regional, national, or even international ones, is not always appropriate; especially when changes to health policy are contemplated. The Jerusalem Study provides an important case study to explore the generalizability of local epidemiological data to other contexts.

The purpose of this commentary is to assess and evaluate this matter.

### Jerusalem as a case study

Epidemiological data collected on a local level are limited to the geographical and sociological circumstances existing in that area. Jerusalem is a striking example for this, even on a micro-geographical level, since the city is home to some hospitals treating either mostly the Arab population or the Jewish Ultra-Orthodox population, depending on their location. Even though the two hospitals included in the Jerusalem Study receive patients from diverse backgrounds, the population in Jerusalem is still quite different from the rest of the country. For example, according to the Israeli Central Bureau of Statistics (CBS), in Jerusalem, Jews account for 61% of the population and Arabs account for 38%. In contrast, on a national level, Jews account for 74% compared to 21% Arabs. In addition, Jerusalem has a bigger proportion of children compared to the rest of the country and has a high concentration of low socio-economic status (SES) communities [1].

These demographic differences are coupled with sociological phenomena that add further complexity. For instance, previous studies demonstrated that Ultra-orthodox Jews and populations with a low SES background are at higher risk for certain types of injury, e.g. burns [2, 3]. This elevated risk could be associated with the unique characteristics of low-SES families, such as increased number of children, sibling supervision, etc. It is reasonable to hypothesize that these sociological phenomena are more prevalent in the Jerusalem area, given its demographic breakdown. Therefore, injury patterns in the Jerusalem area do not necessarily reflect those obtained from national datasets.

### Evaluation of national dataset

In an effort to further explore the applicability of the Jerusalem Study’s findings to the national level, we assessed parallel data from The Israel National Trauma Registry (INTR). The INTR contains data from almost all trauma centers in Israel, including all six Level I Trauma Centers and 14 regional ones. The registry records data regarding all patients admitted to emergency department (ED) due to traumatic injury and subsequently hospitalized with an ICD-9-CM diagnosis codes between 800 and 959.9. The INTR excludes trauma casualties that were not hospitalized, even if admitted to the ED, and those who died outside the hospital. The INTR serves as the national repository for analysis of the epidemiology of injury in Israel [2, 4, 5].

In order to align the data reported in this commentary with that reported in the Jerusalem study, the analysis was restricted to national data from the year 2013. As a preliminary step, in order to explore whether the period assessed for the epidemiology of wrist and hand injury (April–June) was representative of the entire year, we analyzed INTR data for other quartiles of the year. No significant differences were observed in the overall frequency of hand and wrist injuries nor in the frequency of the circumstances of said injury over the months of the year, suggesting that generalization of the Jerusalem study’s data to year-round is plausible.

According to the INTR data, of the 38,881 country-wide hospitalized trauma cases in 2013, about 28% (*n* = 10,776) involved upper extremities injuries. Of those, 3895 patients (36.15%) sustained wrist and hand injuries, making this type of trauma account for 10% of the total trauma cases that year. Geographical comparison of the data suggests that wrist and hand injuries are less frequent in Jerusalem (6.27%) compared to the rest of the country (10.42%), according to Chi-Square test (χ^2^ = 65.170, df = 1, *p* < .0001).

Comparison of injury patterns and circumstances between the Jerusalem Study and the INTR data is challenging, due to differences in categorization. Firstly, the Jerusalem study uses different categories of injury mechanisms and injury types, e.g. by referring to “explosion” as an injury type in contrast to the INTR that views it as an injury mechanism. It should be noted that the methodology section of Luria et al. [4] does list explosion as an injury mechanism, yet the paper includes it under injury types in its “Table 1”. Secondly, the Jerusalem Study used different description of injury circumstances than the INTR, resulting in some being applicable only to the Jerusalem Study, e.g. “ball injury”, “saw/hammer injury”, “door slamming”, etc. Similarly, some of the diagnoses used by the INTR have no apparent equivalents in the Jerusalem Study, such as open wounds (36.71% of INTR cases in 2013), fractures (34.87%), amputations (10.32%), and dislocations or sprains (2.82%).

**Table 1:** Age group distribution in the year 2013 comparing datasets to national statistics by the Central Bureau of Statistics (CBS) and the Israel National Trauma Registry (INTR)

Age group	[4] (Jerusalem)	CBS data (Jerusalem)	CBS data (National data)	INTR (National data)
0–16	34%	43%	34%	23%
17–64	61%	48%	55%	67%
65+	5%	9%	11%	9%

Keeping in mind the above-mentioned challenges in comparing data from the two datasets, there are some remarkable differences between them. For example, the INTR data suggests that in the year 2013, burns accounted for 15% of injury mechanisms among wrist and hand patients in Jerusalem (5% in the rest of the country). In contrast, Luria et al. [6] reported only 2–3% burns in their sample of the Jerusalem population. It is possible that this difference can be attributed to the fact that the Jerusalem study includes all cases admitted to the ED, whereas the INTR data includes only those hospitalized.

Next, the INTR data presents a frequency of 16.5% of superficial contusion among wrist and hand patients, as oppose to 56.1% contusion rate reported in the Jerusalem study. The dramatic difference in contusion frequency might be explained if the Jerusalem study includes fractures in its “contusion” category. Another example of difference is the frequency of hand and wrist injuries caused by falls. While falls account for only 15.9% of said injuries in the INTR data referring to Jerusalem area, the Jerusalem Study reports a frequency of 33.9% of fall-related hand and wrist injuries. However, this difference might be attributed to the fact that fall injuries tend to be less severe, and as a result fewer of these cases are hospitalized.

There are numerous plausible explanations for the observed differences between the results reported by Luria et al. [4] and the INTR data. In addition to those described above, it is also important to note that the INTR data suggests that a considerable portion of wrist and hand injury are caused by work-related accidents (31% nationally, 21% locally in Jerusalem for the year 2013). Since the population in Jerusalem includes more children than other parts of the country (see Table 1), and since far fewer children are exposed to work-related risks, it is reasonable to assume that observed differences in hand and wrist epidemiology might be attributed to this aspect. Other explanations for differences could be attributed to socio-demographic factors, which are not assessed in the INTR, such as religiosity (Jerusalem has a higher proportion of religious population compared to the rest of the country), religion (Jerusalem has a higher proportion of Muslim population), and cultural aspects, e.g. parental supervision of children, attitudes toward risk, etc.

There are also some similarities between the datasets. For instance, the principle injury mechanism in the INTR data was laceration, i.e. cut or stabbing (*n* = 1113; 29.1%), which is comparable with that reported by Luria et al. [4] in the Jerusalem study (26.4%).

The INTR data sheds more light on the epidemiology of hand and wrist injury in Israel during the examined year of 2013 by providing additional information. For instance, after laceration, the most common injury mechanisms were unintentional bruising caused by objects/people (17.51%), transportation-related injury (16.69%), fall (15.89%), and burn (5.80%). The additional 15.02% were other or unknown injury circumstances. The vast majority (85.9%) of cases were lightly wounded casualties (Injury Severity Scale (ISS) of 1–8). Since the Jerusalem Study includes cases discharged from the ED, we can assume that an even bigger portion of its cases were mildly injured.

## Conclusions

The work done by Luria et al. [4] bears significant contribution to the understanding of local epidemiology of hand and wrist injury. Such studies are of important value in surfacing health issues, challenges and phenomena that would otherwise may be missed. Moreover, these studies provide crucial insights into data otherwise not collected and allow for a more accurate identification of risk groups. It has also paramount importance for planning and management of services and programs at the local level, as rightfully suggested in the Jerusalem Study. Luria et al.'s [4] proposal to explore the applicability of their findings to local intervention programs is appropriate. In this commentary we assessed the applicability of the study conducted by Luria et al. [4] on the epidemiology of wrist and hand injuries in Jerusalem to a national setting. It is important to note that all comparisons presented in this commentary were done under serious limitations and should not be taken for face value, rather as demonstrator of qualitative differences between data collected locally and nationally. While the authors do not call for national policy change based on their findings, it is important to state that extrapolating from local epidemiological data obtained from one or two hospitals should be done cautiously due to limitations in generalizing of conclusions. In conclusion, Luria et al’s work is extremely important in generating local epidemiological findings, which in turn should provide the basis for extended research on a national level.

## Data Availability

Data of the Israel National Trauma Registry is protected. Access to data would be considered upon reasonable request.

## Abbreviations

CBS: Central Bureau of Statistics
ED: Emergency Department
INTR: Israel National Trauma Registry
ISS: Injury Severity Score
SES: Socio-Economic Status
